# Supplementary material for: Outer Membrane Vesicles (OMVs) as Biomedical Tools and Their Relevance as Immune-Modulating Agents against H. pylori Infections: Current Status and Future Prospects
Source: Int J Mol Sci. 2023 May 10;24(10):8542. doi: 10.3390/ijms24108542 (PMC10218342; doi:10.3390/ijms24108542)
Supplement: Supplementary file 1 [file ijms-24-08542-s001.zip › ijms-2349024-supplementary.pdf]

Table S1. The models of OMVs biogenesis.

Model	Mode of biogenesis	Ref.
Vesiculation via the VacJ/Yrb ATP-binding cassette ABC transporter	The accumulation of phospholipids in the outer membrane's outer leaflet due to the transcriptional silencing or inactivation of the VacJ/Yrb transporter induces the outward curvature and facilitates OMV formation and release	[1-3]
Insertion of molecules into the outer membrane outer leaflet	The insertion of some molecules into the outer membrane outer leaflet can trigger the outward bulging of the outer membrane and promote OMVs formation	[4-6]
	The insertion of some molecules into the outer membrane outer leaflet (e.g., *Pseudomonas aeruginosa* (*P. aeruginosa*) quinolone signal (PQS)) can trigger the outward bulging of the outer membrane and promote OMVs formation	
Enrichment of some areas of the outer membrane with specific types of LPS, and/or phospholipids	The presence of specific types of LPS, and/or phospholipids enriched areas in the outer membrane allows the vesiculation and OMV release	[7-9]
Accumulation of misfolded proteins, peptidoglycan fragments, and other molecules inside the periplasmic space	The accumulation of these molecules inside the periplasmic space increases the local pressure on the outer membrane that result in OMV formation	[10-13]
Disruption of crosslinks between the peptidoglycan layer and lipoproteins	The disruption of crosslinks between the peptidoglycan layer and lipoproteins allows the outer membrane in these regions to curve and form OMVs	[14-16]

1. Roier, S.; F.G. Zingl; F. Cakar; S. Durakovic; P. Kohl; T.O. Eichmann; L. Klug; B. Gadermaier; K. Weinzerl; R. Prassl; A. Lass; G. Daum; J. Reidl; M.F. Feldman; S. Schild. A novel mechanism for the biogenesis of outer membrane vesicles in Gram-negative bacteria. *Nat. Commun.* **2016**, *7*, 1-13.
2. Zingl, F.G.; P. Kohl; F. Cakar; D.R. Leitner; F. Mitterer; K.E. Bonnington; G.N. Rechberger; M.J. Kuehn; Z. Guan; J. Reidl. Outer membrane vesiculation facilitates surface exchange and in vivo adaptation of *Vibrio cholerae*. *Cell host & microbe* **2020**, *27*, 1-38.
3. Malinverni, J.C.; T.J. Silhavy. An ABC transport system that maintains lipid asymmetry in the gram-negative outer membrane. *Proc. Natl. Acad. Sci.* **2009**, *106*, 8009-8014.
4. Kulp, A.; M.J. Kuehn. Biological functions and biogenesis of secreted bacterial outer membrane vesicles. *Annu. Rev. Microbiol.* **2010**, *64*, 163-184.

5. Tashiro, Y.; S. Ichikawa; T. Nakajima-Kambe; H. Uchiyama; N. Nomura. *Pseudomonas* quinolone signal affects membrane vesicle production in not only Gram-negative but also Gram-positive bacteria. *Microbes Environ.* **2010**, *25*, 120-125.
6. Mashburn-Warren, L.; J. Howe; P. Garidel; W. Richter; F. Steiniger; M. Roessle; K. Brandenburg; M. Whiteley. Interaction of quorum signals with outer membrane lipids: insights into prokaryotic membrane vesicle formation. *Mol. Microbiol.* **2008**, *69*, 491-502.
7. Kadurugamuwa, J.L.; T.J. Beveridge. Virulence factors are released from *Pseudomonas aeruginosa* in association with membrane vesicles during normal growth and exposure to gentamicin: a novel mechanism of enzyme secretion. *J. Bacteriol.* **1995**, *177*, 3998-4008.
8. Sabra, W.; H. Lünsdorf; A.-P. Zeng. Alterations in the formation of lipopolysaccharide and membrane vesicles on the surface of *Pseudomonas aeruginosa* PAO1 under oxygen stress conditions. *Microbiology* **2003**, *149*, 2789-2795.
9. Chowdhury, C.; M.V. Jagannadham. Virulence factors are released in association with outer membrane vesicles of *Pseudomonas syringae* pv. tomato T1 during normal growth. *Biochim. Biophys. Acta - Proteins Proteom.* **2013**, *1834*, 231-239.
10. Schwechheimer, C.; A. Kulp; M.J. Kuehn. Modulation of bacterial outer membrane vesicle production by envelope structure and content. *BMC Microbiol.* **2014**, *14*, 1-13.
11. McBroom, A.J.; M.J. Kuehn. Release of outer membrane vesicles by Gram-negative bacteria is a novel envelope stress response. *Mol. Microbiol.* **2007**, *63*, 545-558.
12. Deatherage, B.L.; B.T. Cookson. Membrane vesicle release in bacteria, eukaryotes, and archaea: a conserved yet underappreciated aspect of microbial life. *Infect. Immun.* **2012**, *80*, 1948-1957.
13. Tashiro, Y.; R. Sakai; M. Toyofuku; I. Sawada; T. Nakajima-Kambe; H. Uchiyama; N. Nomura. Outer membrane machinery and alginate synthesis regulators control membrane vesicle production in *Pseudomonas aeruginosa*. *J. Bacteriol.* **2009**, *191*, 7509-7519.
14. Wensink, J.; B. Witholt. Outer membrane vesicles released by normally growing *Escherichia coli* contain very little lipoprotein. *Eur. J. Biochem.* **1981**, *116*, 331-335.
15. Deatherage, B.L.; J.C. Lara; T. Bergsbaken; S.L. Rassoulian Barrett; S. Lara; B.T. Cookson. Biogenesis of bacterial membrane vesicles. *Mol. Microbiol.* **2009**, *72*, 1395-1407.
16. Toyofuku, M.; G. Cárcamo-Oyarce; T. Yamamoto; F. Eisenstein; C.-C. Hsiao; M. Kurosawa; K. Gademann; M. Pilhofer; N. Nomura; L. Eberl. Prophage-triggered membrane vesicle formation through peptidoglycan damage in *Bacillus subtilis*. *Nat. Commun.* **2017**, *8*, 1-10.
